# Supplementary material for: High Constitutive Cytokine Release by Primary Human Acute Myeloid Leukemia Cells Is Associated with a Specific Intercellular Communication Phenotype
Source: J Clin Med. 2019 Jul 4;8(7):970. doi: 10.3390/jcm8070970 (PMC6678419; doi:10.3390/jcm8070970)
Supplement: Supplementary file 1 [file jcm-08-00970-s001.pdf]

Journal of
Clinical Medicine

MDPI

Supplementary Information

High Constitutive Cytokine Release by Primary Human Acute Myeloid Leukemia Cells Is Associated with a Specific Intercellular Communication Phenotype

Håkon Reikvam [1,2,*], Elise Aasebø [1], Annette K. Brenner [2], Sushma Bartaula-Brevik [1], Ida Sofie Grønningsæter [2], Rakel Brendsdal Forthun [2], Randi Hovland [3,4] and Øystein Bruserud [1,2]

1 Department of Clinical Science, University of Bergen, 5020, Bergen, Norway
2 Department of Medicine, Haukeland University Hospital, 5021, Bergen, Norway
3 Department of Medical Genetics, Haukeland University Hospital, 5021, Bergen, Norway
4 Institute of Biomedicine, University of Bergen, 5020, Bergen, Norway
* Correspondence: Hakon.Reikvam@med.uib.no; Tel.: +55-97-50-00

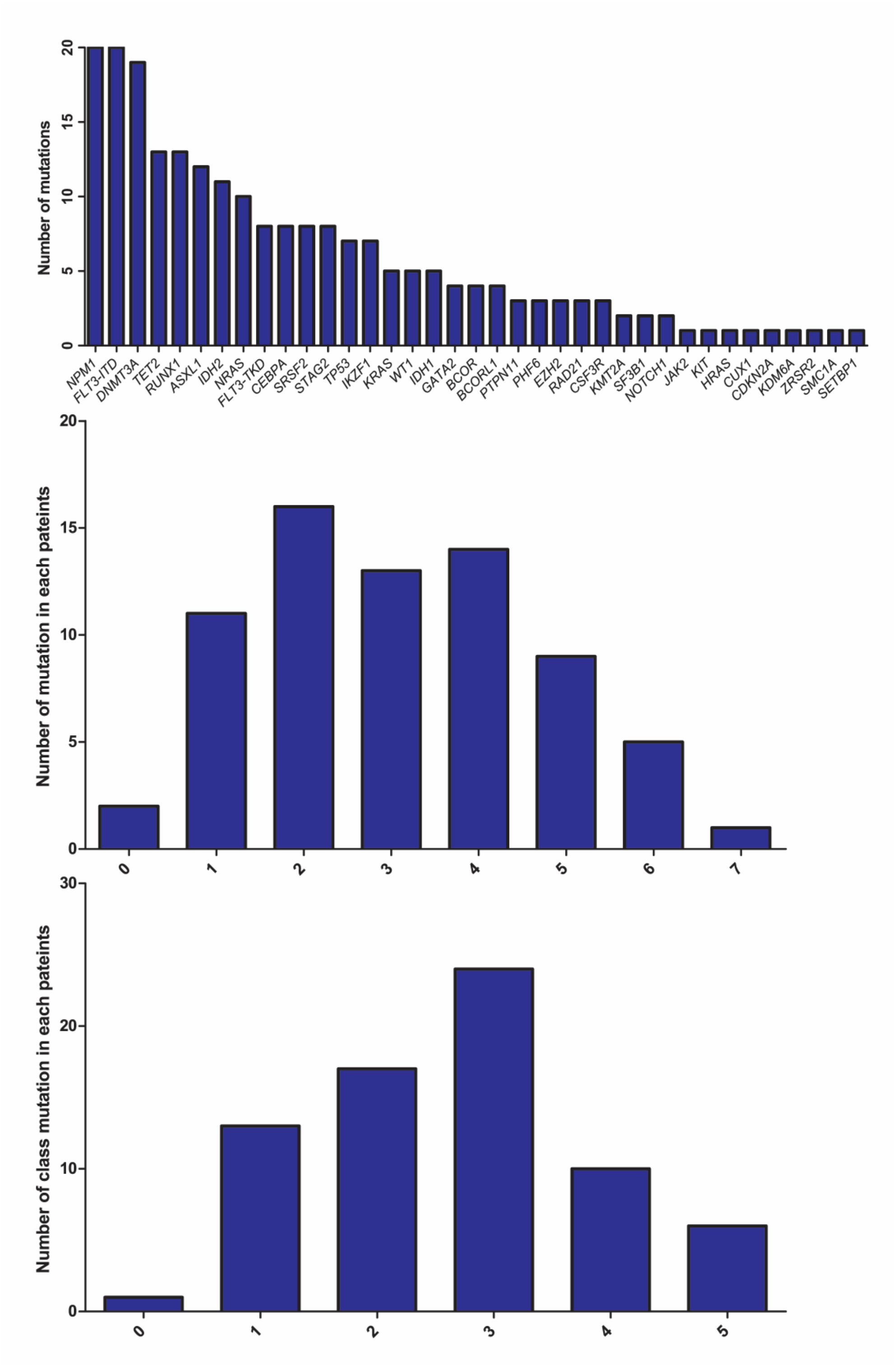

Figure S1. Mutational studies in a cohort of 71 AML patients. The figure shows the number of patients with the various mutations (upper), the number of mutations in for each patient (middle) and the number of main classes with mutation(s) in each patient (lower).

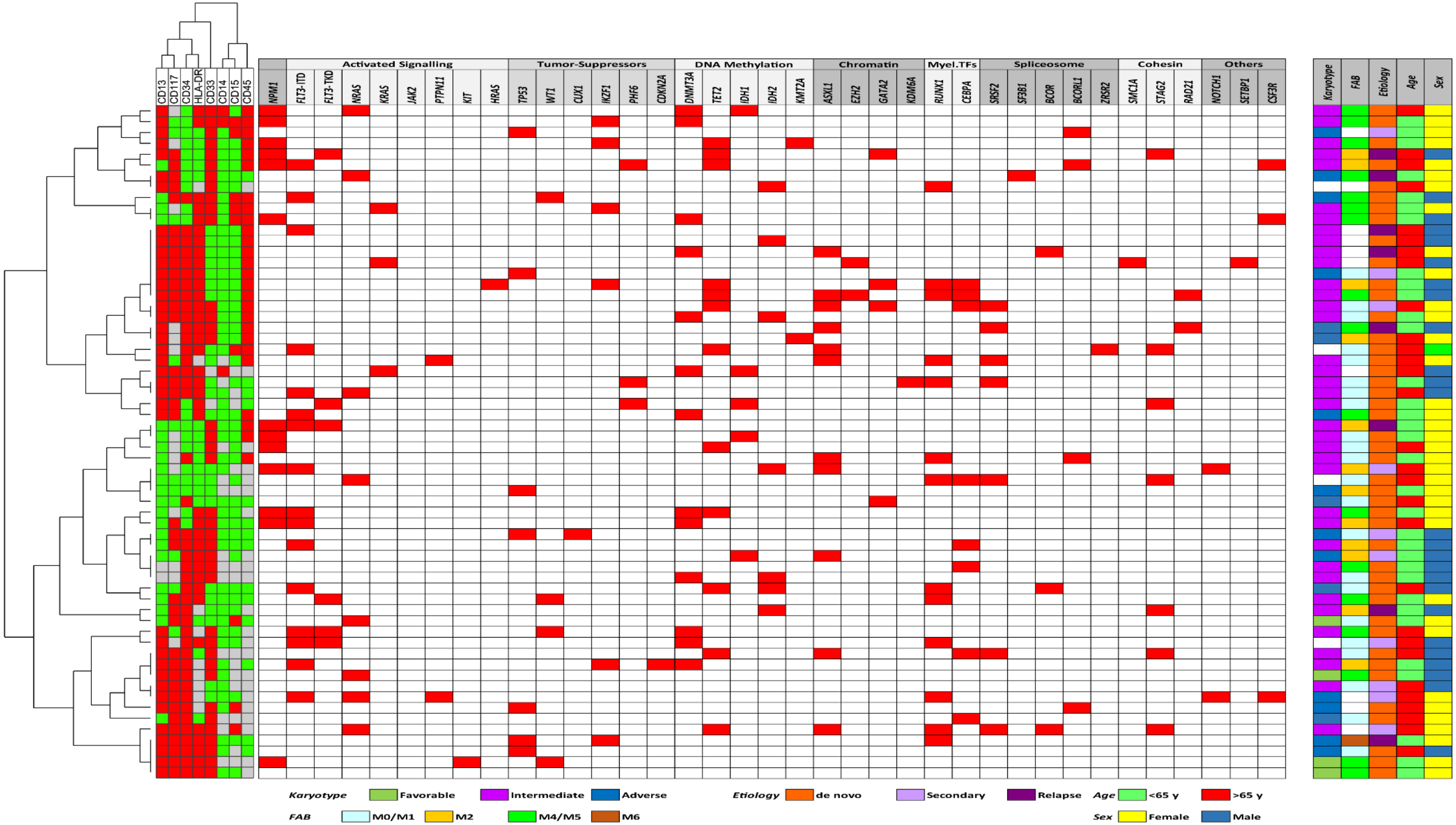

Figure S2. The immunophenotype of primary human AML cells derived from 62 unselected patients. The expression of the eight differentiation markers CD13, CD14, CD15, CD33, CD34, CD45, CD117 and HLA-DR was investigated for 62 of the 71 patients included in our present study. We performed an unsupervised hierarchical cluster analysis and identified four patient main clusters/patient subsets. The mutational profile for each f the 62 patients is also given (middle), no individual mutation of main class of mutations showed any significant association with any of the for differentiation marker clusters (middle). Finally, the four main patient clusters did not differ with regard to karyotype, FAB classification, etiology, age and gender either (right). Grey cells indicate missing data.

We performed a significant analysis of the global gene expression profiles using microarray analysis. We selected discriminative genes by identifying a d-score >3.5 in significance analysis of microarray (SAM), and we could then identify 149 genes being differently expressed between the two groups. We performed an unsupervised hierarchical cluster analysis (Euclidean measure, and complete distance) based on these genes and were able to identify two patient clusters/subsets corresponding to patients with high (red color in the left column) and low constitutive release (blue color); the only outlier was a high-release patient. The two patient subsets did not differ with regard to their mutational profile or their biological or clinical characteristics (karyotype, FAB classification, etiology, age and gender; right part).

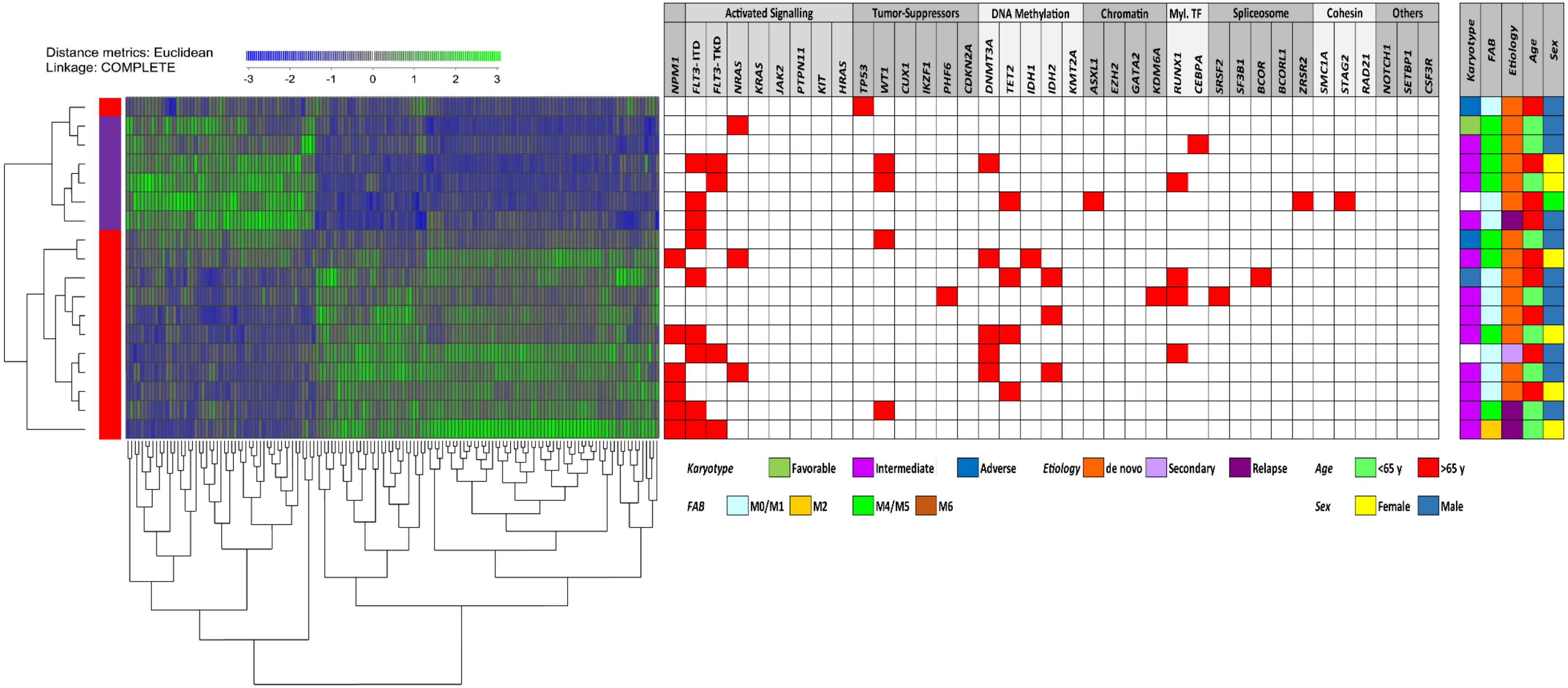

Figure S3. A comparison of global gene expression profiles for unselected patients with high (n = 12 patients) and low (n = 6) constitutive release of extracellular mediators.

Table S1. The biological and clinical characteristics of the 16 patients included in the proteomic studies.

Patients	Sex	Age	Previous	FAB	Hb	TPK	Cell surface markers							Genetic abnormalities		
							CD13	CD14	CD15	CD33	CD34	CD117	HLA-DR	Cytogen	*FLT3*	*NPM1*
**High constitutive release**																
1	M	42		M2	10	63	(+)	-	-	+	+	+	+	Normal	ITD	wt
2	F	66		M1	7.10	80	-	nt	-	+	-	nt	-	Normal	wt	ins
3	M	46		M1	15.40	113	+	nt	+	nt	nt	nt	nt	Normal	wt	ins
4	K	67		M0	8.6	216	(+)	-	nt	+	+	(+)	-	+21	wt	wt
5	M	60		M4	6.1	632	+	-	nt	+	+	+	+	del9	ITD	wt
6	M	53		M0	9.40	84	+	nt	+	+	+	+	+	pluss 13	wt	wt
7	M	67		M0	13.20	180	-	-	-	-	+	-	+	del5	ITD	wt
8	M	62		M4	11.30	8	+	-	-	+	+	+	+	Trisomy 8	wt	wt
**Low constitutive release**																
9	F	18		M4	9.90	46	+	nt	nt	+	+	+	+	Inv16	wt	wt
10	F	46		M1	10.80	1182	+	-	+	+	+	+	+	Inv16	wt	wt
11	F	42		M5	9.20	142	+	+	+	+	-	-	+	Normal	wt	ins
12	M	58		M5	9.60	20	nt	nt	nt	+	+	nt	+	Normal	wt	wt
13	M	48		M5	9.50	17	+	+	-	+	-	+	+	Normal	ITD	ins
14	M	36		M4	9.6	41	nt	(+)	nt	nt	+	+	+	Inv16	wt	wt
15	F	55		M4	11.2	20	+	-	+	+	+	+	+	Inv16	wt	wt
16	F	29	Chemotherapy	M5	8.60	18	+	+	-	+	+	+	+	Normal	ITD+TKD	wt

Table S2. The mutational profile of patients with high or low constitutive mediator release and included in the proteomic studies.

Classification	Mutation	High release group	Low release group	Classification	Mutation	High release group	Low release group
**NPM1**	*NPM1*	2	3	**Chromatin modification**	*ASXL1*	2	0
	**Total group**	2	3		*EZH2*	1	0
**Signaling**	*FLT3*-ITD	3	1		*GATA2*	0	0
	FLT3-TKD	0	2		*KDM6A*	1	0
	HRAS	0	0		**Total group**	4	0
	JAK2	0	0				
	KIT	0	1	**Myeloid transcription factors**	*CEBPA*	3	1
	KRAS	0	0		*RUNX1*	3	1
	NRAS	1	2		**Total group**	6	2
	PTPN11	0	0	**Spliceosome/transcription repressors**	*BCOR*	1	0
	**Total group**	4	6		*BCORL1*	0	0
**Tumor suppressors**	*CDKN2A*	1	0		*SF3B1*	0	0
	CUX1	0	0		*SRSF2*	1	0
	IKZF1	1	1		*ZRSB2*	1	0
	PHF6	1	0		**Total group**	3	0
	TP53	0	0	**Cohesin**	*RAD21*	1	0
	WT1	0	2		*SMC1A*	0	0
	**Total group**	3	3		*STAG2*	1	0
**DNA methylation**	*DNMT3A*	2	2		**Total group**	2	0
	IDH1	0	0	**Others**	*CSF3R*	0	0
	IDH2	2	1		*NOTCH1*	0	0
	KMT2A/MLL	0	0		*SETBP1*	0	0
	TET2	3	0		**Total group**	0	0
	**Total group**	7	3				

Table S3. Statistical comparisons of associations between various mutations and between mutations and signs of AML cell differentiation (morphology, CD34 expression). The analyses are based on Fisher's exact test, two-tailed p-values are given. The table presents the comparison, the corresponding 2x2 tables and the uncorrected p-values. The intention with these analyses was to verify that our present patient population is representative by confirming that we can detect the same significant associations as described in previous larger studies. We then did 11 comparisons and significant p-values are marked in bold; those comparisons that remain significant also when doing a Bonfessoni correction are marked with *.

Comparison	Basis for the statistical analyses			*p*-value
NPM1 mutations versus FLT3-ITD		FLT3-ITD	FLT3 wt	0.4233
	NPM1-mutated	9	11	
	NPM1 wt	11	40	
NPM1 mutations versus DNA metylating gene mutations (DNAmgm)		DNAmgm+	DNAmgm wt	0.0015*
	NPM1-mutated	17	3	
	NPM wt	22	29	
NPM1 mutations versus Myeloid Transcription Factor mutations (MTFm)		MTFm+	MTF wt	<0.0001*
	NPM1-mutated	20	0	
	NPM1 wt	18	33	
TP53 mutations versus complex karyotype		Complex	Not complex	0.049
	TP53 mutated	7	0	
	TP53 wt	10	54	
NPM1 mutation versus morphological differentiation (i.e. FAB classification)		M2/M4/M5/M6	M0/M1	0.0233
	NPM1-mutated	16	3	
	NPM 1 wt	23	21	
NPM1 mutation versus CD33 expression		CD33+	CD33–	0.0208
	NPM1-mutated	12	1	
	NPM1wt	26	21	
NPM1 mutations versus CD34 expression		CD34+	CD34–	<0.0001*
	NPM1-mutated	1	12	
	NPM1 wt	41	8	
FLT3-ITD versus CD34 expression		CD34+	CD34-	0.7522
	FLT3-ITD	10	6	
	FLT3 wt	32	14	
DNMT3A mutations versus CD34 expression		CD34+	CD34-	0.3178
	DNMT3A-mutated	7	6	
	DNMT3A wt	35	14	
Chromatin Modifier (CM) mutations versus CD34 expression		CD34+	CD34-	0.0159
	CM-mutated	28	19	
	CM wt	3	12	

Table 4. Prtoteomic profiling based on AML secretome. The proteomic comparison of enriched primary human AML cells showing high and low constitutive release of extracellular mediators. The results are presented as the protein identity (presented by gene name) together with the z-statistics p-value (z-score) and the Welch's t-test p-value, the median protein levels (of log_2 transformed protein intensities) for AML cells with generally low and high constitutive release of extracellular mediators, and the fold change (in log_2 scale) for each mediator when comparing the level of high constitutive AML cell release cells versus low release cells. A summary of the protein function together with key words is also presented; this information is based on the Gene database and selected references identified in the PubMed database. The selection is based on the GO terms Cell compartment listed in Table 3 in the article (detailed explanation given in the Table, see below).

Protein, Z-score, *p*-value	Level, Low release	Level, High release	Fold change (log_2) High vs. low	Summary	Keywords
**Proteins Showing High Abundance for Cells with Low Constitutive Release** (n = 12)<br>Belonging to the terms GO:0000790-nuclear chromatin and GO:0005736-DNA-directed RNA polymerase complex (see Table 3)					
H1F0<br>$3,46 \times 10^{-10}$<br>0.018516	27.70	25.30	−2.41	*H1 histone family member 0.* Histones are basic nuclear proteins that are responsible for the nucleosome structure of the chromosomal fiber in eukaryotes. Nucleosomes consist of approximately 146 bp of DNA wrapped around a histone octamer composed of pairs of each of the four core histones (H2A, H2B, H3, and H4). The chromatin fiber is further compacted through the interaction of a linker histone, H1, with the DNA between the nucleosomes to form higher order chromatin structures. This gene encodes a replication-independent histone that is a member of the histone H1 family.	Histone,<br>Gene expression
2HIST1H2AJ<br>0.000188<br>0.038121	22.57	21.20	−1.37	*Histone cluster 1 H2A family member j.* Two molecules of each of the four core histones (H2A. H2B. H3. and H4) form an octamer. around which approximately 146 bp of DNA is wrapped in repeating units. called nucleosomes. This gene encodes a replication-dependent histone that is a member of the histone H2A family. This gene is found in the small histone gene cluster on chromosome 6p22-p21.3.	Chromatin<br>Histone
MEN1<br>0.043127<br>0.039881	23.45	22.81	−0,64	Menin 1. This protein is a tumor suppressor. It is a scaffold protein that functions in histone modification and epigenetic gene regulation.	Histone modification<br>Epigenetics
MBD3<br>0.001206<br>0.039855	24.83	23.67	−1.16	*Methyl-CpG binding domain protein 3.*.This gene belongs to a family of nuclear proteins which are characterized by the presence of a methyl-CpG binding domain (MBD). The encoded protein is a subunit of the NuRD. a multisubunit complex containing nucleosome remodeling and histone deacetylase activities. The protein mediates the	Nucleosome<br>Histone deacetylation

				association of metastasis-associated protein 2 with the core histone deacetylase complex.	
JUND<br>0.011365<br>0.007308	23.03	22.17	−0.86	*JunD proto-oncogene, AP-1 transcription factor subunit.* The protein is a member of the JUN family, and a functional component of the AP1 transcription factor complex. This protein has been proposed to protect cells from p53-dependent senescence and apoptosis.	Transcription
POLR1A<br>0.002519<br>0.010079	25.49	24.42	−1.07	*RNA polymerase I subunit A.* The encoded protein is the largest subunit of the RNA polymerase I complex. The encoded protein represents the catalytic subunit of the complex. which transcribes DNA into ribosomal RNA precursors.	Transcription<br>RNA
POLR1B<br>0.003823<br>0.020031	24.10	23.09	−1.02	*RNA polymerase I subunit B.* RNA polymerase I (pol I) is responsible for the transcription of ribosomal RNA (rRNA) genes and production of rRNA. the primary component of ribosomes. Pol I is a multisubunit enzyme. Most of the mass of the pol I complex derives from the 2 largest subunits. Rpa1 and Rpa2 in yeast. POLR1B is homologous to Rpa2	Transcription<br>RNA
POGZ<br>0,001956<br>0,02357	25.14	24.03	−1.10	*Pogo transposable element derived with ZNF domain.* The protein appears to be a zinc finger protein that interacts with the transcription factor SP1.	Transcription
POLR1E (PAF53)<br>0.008485<br>0.049055	23.29	22.39	−0.91	*RNA polymerase I subunit E. Expressed in the bone marrow.* POLR1E/PAF53 is essential for specific rDNA transcription. its levels are regulated in response to growth factors and it is important for cell proliferation (1). Furthermore. nuclear Sirtuin SIRT7 targets POLR1E that is a subunit of RNA polymerase I (Pol I). Acetylation of POLR1E at lysine 373 by CBP and deacetylation by SIRT7 modulate the association of Pol I with DNA. hypoacetylation correlating with increased rDNA occupancy of Pol I and transcription activation. Nucleolar detention requires binding of SIRT7 to nascent pre-rRNA. linking the spatial distribution of SIRT7 and deacetylation of POLR1E to ongoing transcription (2).	Transcription<br>Acetylation
ACTL6A<br>0.017829<br>0.023698	27.85	27.06	−0.79	*Actin like 6A.* This gene encodes an actin-related protein (ARPs). which share significant amino acid sequence identity to conventional actins. The ARPs are involved in vesicular transport. spindle orientation. nuclear migration and chromatin remodeling. This gene encodes a 53 kDa subunit protein of the BAF complex that is thought to facilitate transcriptional activation of specific genes by antagonizing chromatin-mediated transcriptional repression.	Chromatin<br>Tanscription

TWISTNB<br>0.023213<br>0.02204	22.41	21.66	−0.75	*TWIST neighbor.* High bone marrow expression (3).	
SMARCE1<br>0.024052<br>0.032658	27.37	26.63	−0.74	*SWI/SNF related. matrix associated. actin dependent regulator of chromatin. subfamily e. member 1.* The encoded protein is part of the large ATP-dependent chromatin remodeling complex SWI/SNF. which is required for transcriptional activation of genes normally repressed by chromatin. The encoded protein. either alone or when in the SWI/SNF complex. can bind to 4-way junction DNA. which is thought to mimic the topology of DNA as it enters or exits the nucleosome.	Chromatin<br>Transcription<br>Nucleosome
**Proteins Showing High Abundance for Cells with High Constitutive Release** (*n* = 75)<br>These proteins represent the subset of proteins included in the go terms go:0070062~extracellular exosome, go:0005829~cytosol, go:0016020~membrane and go:0005737~cytoplasm (see figure 4 in the article); in addition they were included in the large interacting protein network presented in the left part of Figure 5 in the article.					
**Phagocytosis-neutrophils (*n* = 6)**					
PLXNB2<br>0.032126<br>0.004193	25.00	25.85	0.84	*Plexin B2.* Members of the B class of plexins. such as PLXNB2 are transmembrane receptors that participate cell migration in response to semphorins.	Cell migration
CYBA<br>1.61×10^{-6}<br>0.00	24.70	26.76	2.05	*Cytochrome b-245 alpha chain.* Cytochrome b is comprised of a light chain (alpha) and a heavy chain (beta). This gene encodes the light. alpha subunit which has been proposed as a primary component of the microbicidal oxidase system of phagocytes and the generation of . superoxide.	Phagocytosis<br>Phagocytic vacuole<br>Oxidase
NCF4<br>3.03×10^{-7}<br>0.032204	24.47	26.67	2.20	*Neutrophil cytosolic factor 4.* This protein encoded by this gene is a cytosolic regulatory component of the superoxide-producing phagocyte NADPH-oxidase. This protein is preferentially expressed in cells of myeloid lineage. It interacts primarily with neutrophil cytosolic factor 2 (NCF2/p67-phox) to form a complex with neutrophil cytosolic factor 1 (NCF1/p47-phox). which further interacts with the small G protein RAC1 and translocates to the membrane upon cell stimulation. This complex then activates flavocytochrome b. the membrane-integrated catalytic core of the enzyme system. The PX domain of this protein can bind phospholipid products of the PI(3) kinase. which suggests its role in PI(3) kinase-mediated signaling events. Alternatively spliced transcript variants encoding distinct isoforms have been observed	Phagocytosis<br>PI3K<br>RAC1<br>NADPH Oxydase
NCF2	25.45	27.82	2.37	*Neutrophil cytosolic factor 2.* Cytosolic factor 2 is the 67-kilodalton	Neutrophils

3.46×10^{-8}<br>0.033195				cytosolic subunit of the multi-protein NADPH oxidase complex found in neutrophils. This oxidase produces a burst of superoxide which is delivered to the lumen of the neutrophil phagosome. Alternative splicing results in multiple transcript variants encoding different isoforms.	Phagocytosis<br>Phagocytic vacuoles<br>Oxidase
MNDA<br>0<br>0.013294	27.17	30.74	3.57	*Myeloid cell nuclear differentiation antigen.* The protein is detected only in nuclei of cells of the granulocyte-monocyte lineage. MNDA resembles IFI16, suggesting that these genes participate in blood cell-specific responses to interferons.	Differentiation<br>Neutrophil<br>Monocyte
NCF1<br>0<br>0.008745	23.81	28.24	4.43	*Neutrophil cytosolic factor 1.*	Neutrophil
			**Intracellular Signalling (*n* = 15)**		
SYK<br>0.014238<br>0.028254	27.18	28.17	0.99	*Spleen associated tyrosine kinase.* The protein is a non-receptor type Tyr protein kinase widely expressed in hematopoietic cells and involved in coupling activated immunoreceptors to downstream signaling events that mediate diverse cellular responses. including proliferation. differentiation. and phagocytosis. Alternatively spliced transcript variants encoding different isoforms have been found for this gene.	Intracellular signalling<br>Non-receptor tyrosine kinase
ITGAL<br>0.03177<br>0.011871	26.77	27.61	0.84	*Integrin subunit alpha L.* The encoded integrin alpha L chain forms a heterodimeric membrane proteins with the beta 2 chain (ITGB2. see below). The ligands are ICAMs 1-3 (intercellular adhesion molecules 1 through 3). Two transcript variants encoding different isoforms have been found for this gene.	Integrin alpha chain<br>ITGB2
ITGAL<br>0.001299<br>0.016526	20.76	22.11	1.34		Integrin alpha chain
ITGAM<br>9.7E-14<br>0.026935	25.54	28.76	3.22	*Integrin subunit alpha M.* This integrin alpha M chain forms a heterodimeric membrane proteins with the beta 2 chain (ITGB2. see below) to form a leukocyte-specific integrin referred to as macrophage receptor 1 ('Mac-1'). or inactivated-C3b (iC3b) receptor 3 ('CR3'). This heterodimer is important in the adherence of neutrophils and monocytes to stimulated endothelium. and also in the phagocytosis of complement coated particles. Multiple isoforms have been found.	Integrin alpha chain<br>ITGB2
ITGB2	28.57	30.09	1.53	*Integrin subunit beta 2.* This gene encodes an integrin beta chain. which	Integrin beta

0.000299<br>0.017857				combines with multiple different alpha chains to form different integrin heterodimers. The encoded protein plays an important role in immune response and defects in this gene cause leukocyte adhesion deficiency. Alternative splicing results in multiple transcript variants.	chain<br>ITGAL<br>ITGAM
HCLS1<br>0.028429<br>0.001312	28.22	29.08	0.87	*Hematopoietic cell-specific Lyn substrate 1.* High bone marrow expression.	SRC tyrosine kinase family<br>LYN kinase
CBL<br>0.004201<br>0.023884	24.55	25.73	1.18	*Cbl proto-oncogene.* This gene is a proto-oncogene that encodes a RING finger E3 ubiquitin ligase. The encoded protein is one of the enzymes required for targeting substrates for degradation by the proteasome. This protein mediates the transfer of ubiquitin from ubiquitin conjugating enzymes (E2) to specific substrates. This protein also contains an N-terminal phosphotyrosine binding domain that allows it to interact with numerous tyrosine-phosphorylated substrates and target them for proteasome degradation. As such it functions as a negative regulator of many signal transduction pathways. This gene has been found to be mutated or translocated in many cancers including AML	Proto-oncogene<br>Ubiquitin ligase<br>AML
LILRB2<br>1.1×10^{-6}<br>0.005469	22.15	24.24	2.09	*Leukocyte immunoglobulin like receptor B2.* The encoded protein belongs to the subfamily B class of LIR receptors which contain two or four extracellular immunoglobulin domains. a transmembrane domain. and two to four cytoplasmic immunoreceptor tyrosine-based inhibitory motifs (ITIMs). The receptor binds to MHC class I molecules. Multiple transcript variants encoding different isoforms have been found for this gene.	HLA class I
FGR<br>2.21×10^{-8}<br>0.030902	23.77	26.18	2.41	*FGR proto-oncogene. Src family tyrosine kinase.* This gene is a member of the Src family of protein tyrosine kinases. The encoded protein contains N-terminal sites for myristylation and palmitylation. a PTK domain. and SH2 and SH3 domains which are involved in mediating protein-protein interactions with phosphotyrosine-containing and proline-rich motifs. respectively. The protein localizes to plasma membrane ruffles. and functions as a regulator of cell migration and adhesion triggered by the beta-2 integrin signal transduction pathway. Multiple alternatively spliced variants have been identified.	SRC family tyrosine kinase<br>ITGB2
HCK<br>3.64×10^{-11}<br>0.037511	23.48	26.34	2.86	*HCK proto-oncogene. Src family tyrosine kinase.* The protein is a member of the Src family of tyrosine kinases. It is primarily hemopoietic. particularly in cells of the myeloid lineages. It may help couple the Fc	SRC tyrosine kinase family<br>Fc receptors

				receptor to the activation of the respiratory burst. In addition. it may play a role in migration. Multiple isoforms with different subcellular distributions are produced.	Respiratory burst Migration
AGTRAP<br>3.95×10^{-8}<br>0.001303	23.57	25.93	2.36	*Angiotensin II receptor associated protein.* The encoded transmembrane protein localized to the plasma membrane and perinuclear vesicular structures. It interacts with the angiotensin II type I receptor and negatively regulates angiotensin II signaling. Renin-Angiotensin may be important in AML (4).	Intracellular signaling
ANXA2;ANXA2P2<br>0.000409<br>0.002328	31.17	32.66	1.49	*Annexin A2.* This is a member of the annexin family that is a family of calcium-dependent phospholipid-binding proteins that play a role in cellular growth and in signal transduction. This protein functions as an autocrine factor.	Signaling Proliferation
CECR1/ADA2<br>1×10^{-5}<br>0.013525	25.75	27.64	1.89	*Adenosine deaminase 2.* This gene encodes a member of a subfamily of the adenosine deaminase protein family. The encoded protein regulates levels of the signaling molecule adenosine, is secreted and may regulate cell proliferation and differentiation.	Signaling Adenosin Differentiation
INPP5D<br>0.047215<br>0.044399	27.20	27.97	0.76	*Inositol polyphosphate-5-phosphatase D.* The gene is a member of the inositol polyphosphate-5-phosphatase (INPP5) family. It encodes a protein whose expression is restricted to hematopoietic cells where its movement from the cytosol to the plasma membrane is mediated by tyrosine phosphorylation. At the plasma membrane, the protein hydrolyzes the 5' phosphate from phosphatidylinositol (3,4,5)-trisphosphate and inositol-1,3,4,5-tetrakisphosphate, thereby affecting multiple signaling pathways. The protein is also partly localized to the nucleus, where it may be involved in nuclear inositol phosphate signaling processes. Overall, the protein functions as a regulator of myeloid cell proliferation and survival.	Signaling
LPXN<br>0.007032<br>0.025493	24.69	25.79	1.10	*Leupaxin.* The protein is preferentially expressed in hematopoietic cells and belongs to the paxillin family, a focal-adhesion-associated adaptor-protein family. It may function in cell type-specific signaling by associating with PYK2, a member of focal adhesion kinase family. The protein may also function in tyrosine kinase activity.	Signaling Fokal adhesion kinase
**Cytoskeleton. Intracellular trafficking. Cell adhesion (*n* = 24)**					
ACTR3<br>0.046977<br>0.029739	30.03	30.79	0.77	*Actin related protein 3.*	

VAMP3<br>0.046301<br>0.003763	25.18	25.95	0.77	*Vesicle associated membrane protein 3.* Synaptobrevins/VAMPs. syntaxins. and the 25-kD synaptosomal-associated protein are the main components of a protein complex involved in the docking and/or fusion of synaptic vesicles with the presynaptic membrane. This gene is a member of the vesicle-associated membrane protein (VAMP)/synaptobrevin family.	Fusion of vesicles to the surface membrane
DNM2<br>0.041668<br>0.016485	21.34	22.13	0.79	*Dynamin 2.* Dynamins represent one of the subfamilies of GTP-binding proteins. These proteins share considerable sequence similarity in the GTPase domain. Dynamins are associated with microtubules. They have been implicated in cell processes such as endocytosis and cell motility. Dynamins bind many proteins that bind actin and other cytoskeletal proteins. Dynamins can also self-assemble. a process that stimulates GTPase activity. Five alternatively spliced transcripts have been described.	GTPase<br>Microtubule<br>Cytoskeleton<br>Endocytosis<br>Motility
EPN1<br>0.040906<br>0.006474	22.96	23.76	0.79	*Sarcospan.* This is a member of the dystrophin-glycoprotein complex (DGC) is comprised of dystrophin. syntrophin. alpha- and beta-dystroglycans and sarcoglycans. The DGC provides a structural link between the cytoskeleton and the extracellular matrix. Two transcript variants have been described.	Cytoskeleton<br>Extracellular matrix
SH3KBP1<br>0.015021<br>0.002592	25.69	26.67	0.98	*SH3 domain containing kinase binding protein 1.* This gene encodes an adapter protein that contains one or more N-terminal Src homology domains. a proline rich region and a C-terminal coiled-coil domain. The encoded protein facilitates protein-protein interactions and has been implicated in numerous cellular processes including apoptosis. cytoskeletal rearrangement. cell adhesion and in the regulation of clathrin-dependent endocytosis. Alternate splicing results in multiple transcript variants.	Adapter protein.<br>Cytoskeleton<br>Cell adheseion<br>Apoptosis
PICALM<br>0.011976<br>0.00578	25.79	26.80	1.02	*Phosphatidylinositol binding clathrin assembly protein.* This gene encodes a clathrin assembly protein. which recruits clathrin and adaptor protein complex 2 (AP2) to cell membranes at sites of coated-pit formation and clathrin-vesicle assembly. The protein is involved in AP2-dependent clathrin-mediated endocytosis. A chromosomal translocation t(10;11)(p13;q14) leading to the fusion of this gene and the MLLT10 gene is found in acute myeloid leukemia. Multiple spliced variants have been detected.	Clatrin vesicles<br>AML
ARRB2<br>0.001072	25.42	26.79	1.37	*Arrestin beta 2.* Members of arrestin/beta-arrestin protein family are thought to participate in agonist-mediated desensitization of G-	G-protein coupled receptors.

0.01648				protein-coupled receptors. Multiple alternatively spliced transcript variants have been found.	
SNX18<br>5.58×10^{-5}<br>0.010733	21.86	23.57	1.71	*Sorting nexin 18.* This gene encodes a member of the sorting nexin family. Members of this family contain a phox (PX) domain. which is a phosphoinositide binding domain. and are involved in intracellular trafficking. Multiple isoforms have been found.	Intracellular trafficking
AHNAK<br>0.009867<br>0.011693	31.88	32.93	1.05	*AHNAK nucleoprotein.* The protein is a structural scaffold protein that may play a role in cell structure and migration. A shorter variant initiates a feedback loop that regulates alternative splicing of this gene.	Structure<br>Migration
AP1G2<br>0.0014<br>0.010375	23.24	24.57	1.33	*Adaptor related protein complex 1 subunit gamma 2.* Adaptins, together with medium and small subunits, form a heterotetrameric complex called an adaptor, whose role is to promote the formation of clathrin-coated pits and vesicles. The protein encoded by this gene is a gamma-adaptin and belongs to the adaptor complexes large subunits family. It is thought to function at a trafficking step in the pathways between the trans-Golgi network and the cell surface.	Intracellular trafficking
S100A10<br>0.001101<br>0.016931	26.24	27.61	1.37	*S100 calcium binding protein A10.* The protein is a member of the S100 family of calcium-binding proteins. S100 proteins are localized in the cytoplasm and/or nucleus and are involved in regulation of cell cycle progression and differentiation. This protein may function in exocytosis and endocytosis.	Trafficking<br>Exocytosis<br>Endocytosis
S100A4<br>0.013936<br>0.021936	29.66	30.65	0.99	*S100 calcium binding protein A4.* This member of the S100 family of proteins seems to function in motility, invasion, and tubulin polymerization.	Trafficking<br>Cytoskeleton
TOM1<br>0.007641<br>0.021214	25.53	26.62	1.09	*Target of myb1 membrane trafficking protein.* The encoded protein shares its N-terminal domain in common with proteins associated with vesicular trafficking at the endosome. It is recruited to the endosomes by its interaction with endofin.	Trafficking<br>Endosomes
SDCBP<br>1.76×10^{-5}<br>0.01435	23.04	24.87	1.83	*Syndecan binding protein.* This transmembrane protein links syndecan-mediated signaling to the cytoskeleton. This protein may also affect cytoskeletal-membrane organization, cell adhesion, protein trafficking, and the activation of transcription factors. It is also found at the endoplasmic reticulum and nucleus.	Cytoskeleton<br>Signaling<br>Endoplasmatic reticulum
CKAP4<br>1.41×10^{-7}	24.79	27.05	2.26	*Cytoskeleton associated protein 4. High expression in bone marrow cells.*	Cytoskelton

0.027987					
FAM49B<br>0.003202<br>0.027396	26.95	28.17	1.22	*Family with sequence similarity 49 member B. Broad expression in bone marrow.* A regulator of annexin and of mitochondrial functions (5, 6).	Cytoskeleton<br>Mitochondria
CTSH<br>1.93 × 10^{-14}<br>0.035829	22.34	25.65	3.31	*Cathepsin H.* This lysosomal cysteine proteinase is important in degradation of lysosomal proteins. It is an aminopeptidase and an endopeptidase. Increased expression has been correlated with malignant progression of prostate tumors.	Lysosome<br>Peptidase<br>Carcinogenesis
CTSS<br>1.94E-05<br>0.008482	26.11	27.93	1.82	*Cathepsin S.* This is a lysosomal cysteine proteinase.	Lysosome
CTSZ<br>0.001692<br>0.001369	26.66	27.97	1.31	*Cathepsin Z.* The encoded protein is lysosomal cysteine proteinase. This gene is expressed ubiquitously in cancer cell lines and primary tumors.	Lysosome<br>Proteinase
LYZ<br>0<br>0.015745	29.17	33.40	4.22	*Lysozyme.* This protein is found in lysosomes.	Lysosomes
PSAP<br>0.02298<br>0.038796	28.04	28.94	0.90	*Prosaposin.* This protein is proteolytically processed to generate four main cleavage products including saposins A, B, C, and D. Saposins A-D localize primarily to the lysosomal compartment where they facilitate the catabolism of glycosphingolipids. The precursor protein exists both as a secretory protein and as an integral membrane protein.	Lysosome<br>Extracellular
DNAJC13<br>0.01467<br>0.038713	26.57	27.56	0.98	*DnaJ heat shock protein family (Hsp40) member C13.* This member of the Dnaj protein family whose members act as co-chaperones of a partner heat-shock protein. It associates with the heat-shock protein Hsc70 and plays a role in clathrin-mediated endocytosis.	Endocytosis<br>Chaperon
EPN1<br>0.040906<br>0.006474	22.96	23.76	0.79	*Epsin 1.* This epsin binds to clathrin and is involved in the endocytosis of clathrin-coated vesicles. Can be involved in carcinogenesis.	Endocytosis<br>Clathrin<br>Carcinogenesis
			**RAC family-GTPases-Toll Like Receptors (*n* = 25)**		
RAC1<br>0.029868<br>0.041908	25.46	26.32	0.86	*Rac family small GTPase 1.* The protein encoded by this gene is a GTPase which belongs to the RAS superfamily of small GTP-binding proteins. Members of this superfamily appear to regulate a diverse array of cellular events. including the control of cell growth. cytoskeletal reorganization. and the activation of protein kinases. Two	GTPase<br>Cytoskeleton<br>Kinase activation

				transcript variants encoding different isoforms have been found for this gene.	
RHOT1<br>0.046607<br>0.02986	24.17	24.93	0.77	*Ras homolog family member T1.*	Ras homolog
ARHGEF1<br>0.037731<br>0.024791	26.61	27.42	0.81	*Rho guanine nucleotide exchange factor 1.* Rho GTPases play a fundamental role in numerous cellular processes that are initiated by extracellular stimuli that work through G protein coupled receptors (GPCR). The encoded protein may form complex with G proteins and stimulate Rho-dependent signals. Multiple alternatively spliced transcript variants have been found.	GTPase<br>GPCR<br>Rho dependent signals
ARHGAP9<br>0.022128<br>0.027527	24.58	25.49	0.91	*Rho GTPase activating protein 9.* This gene encodes a member of the Rho-GAP family of GTPase activating proteins. The protein has substantial GAP activity towards several Rho-family GTPases in vitro. converting them to an inactive GDP-bound state. It is implicated in regulating adhesion of hematopoietic cells to the extracellular matrix. Multiple transcript variants have been found.	Rho-GAP family<br>GTPase<br>Adhesion
IL16<br>0.021885<br>0.040415	26.21	27.12	0.91	*Interleukin 16. This is* a pleiotropic cytokine that can function as a chemoattractant. The signaling process of this cytokine is mediated by CD4. The cytokine function is attributed to the secreted C-terminal peptide. while the N-terminal product may play a role in cell cycle control. Multiple transcript variants have been found.	Cytokine<br>Cell cycle regulation
FMNL1<br>0.020268<br>0.012477	27.29	28.22	0.93	*Formin like 1.* This gene encodes a formin-related protein; these proteins have been implicated in morphogenesis. cytokinesis. and cell polarity. An alternative splice variant has been described.	Cytokinesis
PKN1<br>0.011951<br>0.045995	24.62	25.64	1.02	*Protein kinase N1.* The protein belongs to the protein kinase C superfamily. This kinase is activated by Rho family of small G proteins and may mediate the Rho-dependent signaling pathway. This kinase can be activated by phospholipids and by limited proteolysis. The 3-phosphoinositide dependent protein kinase-1 (PDPK1/PDK1) is reported to phosphorylate this kinase. which may mediate insulin signals to the actin cytoskeleton. The proteolytic activation of this kinase by caspase-3 or related proteases during apoptosis suggests its role in signal transduction of apoptosis. Alternatively spliced variants have been found.	PKC family kinase<br>Rho family<br>GTPase<br>PDK1<br>Insulin<br>Cytoskeleton<br>Apoptosis
RHOG	27.40	28.49	1.09	*Ras homolog family member G.* This gene encodes a member of the Rho	Rho family

0.007631<br>0.023656				family of small GTPases. which cycle between inactive GDP-bound and active GTP-bound states and function as molecular switches in signal transduction cascades. Rho proteins promote reorganization of the actin cytoskeleton. The encoded protein facilitates translocation of a functional guanine nucleotide exchange factor (GEF) complex from the cytoplasm to the plasma membrane where ras-related C3 botulinum toxin substrate 1 is activated to promote lamellipodium formation and cell migration.	GTPase<br>Cytoskeleton<br>Cell migration
ARAP1<br>0.006072<br>0.01367	25.03	26.15	1.13	*ArfGAP with RhoGAP domain. ankyrin repeat and PH domain 1.* The protein contains SAM. ARF-GAP. RHO-GAP. ankyrin repeat. RAS-associating. and pleckstrin homology (PH) domains. In vitro. this protein displays RHO-GAP and phosphatidylinositol (3.4.5) trisphosphate (PIP3)-dependent ARF-GAP activity. The encoded protein associates with the Golgi. and the ARF-GAP activity mediates changes in the Golgi and the formation of filopodia. It is thought to regulate the cell-specific trafficking of a receptor protein involved in apoptosis. Multiple transcript variants have been found.	Golgi<br>Cell trafficking<br>Apoptosis
ARHGAP30<br>0.000493<br>0.006914	23.15	24.61	1.47	*Rho GTPase activating protein 30.* Broad expression in normal bone marrow. spleen and lymphoid organs.	Rho GTPase
PREX1<br>0.000316<br>0.041008	24.06	25.58	1.52	*Phosphatidylinositol-3.4.5-trisphosphate dependent Rac exchange factor 1.* The protein encoded by this gene acts as a guanine nucleotide exchange factor for the RHO family of small GTP-binding proteins (RACs). It has been shown to bind to and activate RAC1 by exchanging bound GDP for free GTP. The encoded protein. which is found mainly in the cytoplasm. is activated by phosphatidylinositol-3.4.5-trisphosphate and the beta-gamma subunits of heterotrimeric G proteins.	Rho family<br>G protein<br>GTPase
GMIP<br>5.67×10^{-5}<br>0.022388	24.14	25.85	1.71	*GEM interacting protein.* This is a ARHGAP family of Rho/Rac/Cdc42-like GTPase activating protein. The encoded protein interacts with the Ras-related protein Gem through its N-terminal domain. Separately. it interacts with RhoA through a RhoGAP domain. and stimulates RhoA-dependent GTPase activity. Multiple transcript variants have been found.	Rho family<br>GTPase<br>RhoA
DOK2<br>2.57×10^{-6}<br>0.026624	23.41	25.42	2.01	*Docking protein 2.* The protein is constitutively tyrosine phosphorylated in hematopoietic progenitors isolated from chronic myelogenous leukemia (CML) patients in the chronic phase. It may be	Ras/GAP<br>CML

				a critical substrate for p210(bcr/abl). This encoded protein binds p120 (RasGAP) from CML cells.	
ELMO2<br>4.04×10^{-8}<br>9.89×10^{-5}	22.43	24.79	2.36	*Engulfment and cell motility 2.* This protein interacts with the dedicator of cyto-kinesis 1 protein; it is possibly important in phagocytosis and cell migration. Alternative splicing results in multiple transcript variants.	Phagocytosis<br>Cell migration
AKAP13<br>1.11×10^{-8}<br>0.001665	22.96	25.42	2.46	*A-kinase anchoring protein 13.* The A-kinase anchor proteins (AKAPs) bind to the regulatory subunit of protein kinase A (PKA) and confining the holoenzyme to discrete locations within the cell. This gene encodes a member of the AKAP family. Alternative splicing of this gene results in multiple transcripts. The DH domain is associated with guanine nucleotide exchange activation for the Rho/Rac family of small GTP binding proteins. resulting in the conversion of the inactive GTPase to the active form capable of transducing signals. Therefore. these isoforms function as scaffolding proteins to coordinate a Rho signaling pathway and function as protein kinase A-anchoring proteins.	Rho/Rac family<br>GTPase<br>Protein kinase A
ARHGAP27<br>0.035273<br>0.01106	22.36	23.18	0.82	*Rho GTPase activating protein 27.* This protein is a member of a large family of proteins that activate Rho-type guanosine triphosphate (GTP) metabolizing enzymes. It may pay a role in clathrin-mediated endocytosis.	GTPase<br>Endocytosis
GPSM3<br>0.001209<br>0.007015	23.05	24.40	1.35	*G protein signaling modulator 3.* Very high expression in normal bone marrow.	Signaling<br>G protein
GRK6<br>0.004294<br>0.024724	22.74	23.92	1.18	*G protein-coupled receptor kinase 6.* The encoded protein is a member of the G protein-coupled receptor kinase subfamily of the Ser/Thr protein kinase family. The protein phosphorylates the activated forms of G protein-coupled receptors thus initiating their deactivation.	Signaling<br>G protein
IQGAP1<br>4.72×10^{-5}<br>0.003266	29.34	31.07	1.73	*Q motif containing GTPase activating protein 1.* This protein contains four IQ domains, one calponin homology domain, one Ras-GAP domain and one WW domain. It interacts with components of the cytoskeleton, with cell adhesion molecules, and with several signaling molecules to regulate cell morphology and motility.	GTPase<br>Cytoskeleton<br>Sigaling
RAB27A<br>0.001729<br>0.033639	26.08	27.38	1.31	*RAB27A, member RAS oncogene family.* The protein belongs to the small GTPase superfamily, Rab family. The protein is membrane-bound and may be involved in protein transport and small GTPase mediated	GTPase<br>Trafficking<br>Signaling

				signal transduction.	
TLR2<br>3.6×10^{-12}<br>0.007197	23.12	26.12	3.00	*Toll like receptor 2*. This cell-surface protein can form heterodimers with other TLR family members to recognize conserved molecules. Activation of TLRs by PAMPs leads to up-regulated signaling pathways. This protein is also thought to promote apoptosis in response to bacterial lipoproteins. Alternative splicing results in multiple transcript variants.	TLR<br>Intracellular signaling<br>Apoptosis
TOLLIP<br>0.02023<br>0.019808	24.21	25.13	0.93	*Toll interacting protein*. This ubiquitin-binding protein interacts with several Toll-like receptor (TLR) signaling cascade components. The encoded protein regulates inflammatory signaling and is involved in interleukin-1 receptor trafficking and in the turnover of IL1R-associated kinase. Several isoforms have been found.	TLR<br>NFκB<br>IL1 receptor<br>Intracellular trafficking
NFKB1<br>0.025365<br>0.006843	25.67	26.56	0.89	*Nuclear factor kappa B subunit 1*. This gene encodes a 105 kD protein which can undergo cotranslational processing by the 26S proteasome to produce a 50 kD protein. The 105 kD protein is a Rel protein-specific transcription inhibitor and the 50 kD protein is a DNA binding subunit of the NF-kappa-B (NFKB) protein complex. Activated NFKB translocates into the nucleus and stimulates gene expression. Alternative splicing results in multiple isoforms.	NFκB<br>Transcription
IKBKG<br>0.045093<br>0.031763	23.53	24.31	0.77	*Inhibitor of nuclear factor kappa B kinase regulatory subunit gamma*. This encoded protein is a regulatory subunit of the inhibitor of kappaB kinase (IKK) complex, which activates NF-kappaB resulting in activation of gene expression.	NFκB<br>Transcription<br>Signaling
TBK1<br>0.031434<br>0.040984	22.65	23.50	0.85	*TANK binding kinase 1*. The NF-kappa-B (NFKB) complex of proteins is inhibited by I-kappa-B (IKB) proteins. Phosphorylation of serine residues on the IKB proteins by IKB kinases marks them for destruction via the ubiquitination pathway. This protein is similar to IKB kinases and can mediate NFKB activation.	NFκB<br>Signaling<br>Phosphorylation
			**Others (*n* = 5)**		
DBNL<br>0.027079<br>0.021184	26.89	27.76	0.87	*Drebrin like*. Expressed in bone marrow. Pro-oncogenic function (7).	Oncogenic function
FTL<br>4.07×10^{-6}<br>0.025378	27.49	29.46	1.97	*Ferritin light chain*. This gene encodes the light subunit of the ferritin protein, the major intracellular iron storage protein.	Iron metabolism
IMPDH1	24.04	25.74	1.71	*Inosine monophosphate dehydrogenase 1*. The protein acts as a	Nucleotide

5.78×10^{-5}<br>0.000292				homotetramer to regulate cell growth. It catalyzes the synthesis of xanthine monophosphate (XMP) from inosine-5'-monophosphate (IMP). This is the rate-limiting step in the de novo synthesis of guanine nucleotides.	synthesis or metabolism
PDXK<br>0.000175<br>0.042089	24.94	26.53	1.59	*Pyridoxal kinase.* The protein phosphorylates vitamin B6, a step required for the conversion of vitamin B6 to pyridoxal-5-phosphate, an important cofactor in intermediary metabolism.	Vitamin metabolism
SERPINA1<br>0.000257<br>0.037304	27.54	29.08	1.54	*Serpin family E member 1.* This serine proteinase inhibitor is the principal inhibitor of tissue plasminogen activator (tPA) and urokinase (uPA).	Protease Coagulation

References

1. Rothblum, L.I.; Rothblum, K.; Chang, E. PAF53 is essential in mammalian cells: CRISPR/Cas9 fails to eliminate PAF53 expression. *Gene* **2017**, *612*, 55–60.
2. Chen, S.; Seiler, J.; Santiago-Reichelt, M.; Felbel, K.; Grummt, I.; Voit, R. Repression of RNA polymerase I upon stress is caused by inhibition of RNA-dependent deacetylation of PAF53 by SIRT7. *Mol. Cell*. **2013**, *52*, 303–313.
3. Kosan, C.; Kunz, J. Identification and characterisation of the gene TWIST NEIGHBOR (TWISTNB) located in the microdeletion syndrome 7p21 region. *Cytogenet*. *Genome Res*. **2002**, *97*, 167–170.
4. Beyazit, Y.; Aksu, S.; Haznedaroglu, I.C.; Kekilli, M.; Misirlioglu, M.; Tuncer, S.; Karakaya, J.; Koca, E.; Buyukasik, Y.; Sayinalp, N.; et al. Overexpression of the local bone marrow renin-angiotensin system in acute myeloid leukemia. *J. Natl. Med. Assoc.* **2007**, *99*, 57–63.
5. Shang, W.; Jiang, Y.; Boettcher, M.; Ding, K.; Mollenauer, M.; Liu, Z.; Wen, X.; Liu, C.; Hao, P.; Zhao, S.; et al. Genome-wide CRISPR screen identifies FAM49B as a key regulator of actin dynamics and T cell activation. *Proc. Natl. Acad. Sci. USA* **2018**, *115,* E4051–E60.
6. Chattaragada, M.S.; Riganti, C.; Sassoe, M.; Principe, M.; Santamorena, M.M.; Roux, C.; Curcio, C.; Evangelista, A.; Allavena, P.; Salvia, R.; et al. FAM49B, a novel regulator of mitochondrial function and integrity that suppresses tumor metastasis. *Oncogene* **2018**, *37*, 697–709.
7. Li, Z.; Park, H.R.; Shi, Z.; Li, Z.; Pham, C.D.; Du, Y.; Khuri,F.R.; Zhang, Y.; Han, Q.; Fu, H. Pro-oncogenic function of HIP-55/Drebrin-like (DBNL) through Ser269/Thr291-phospho-sensor motifs. *Oncotarget* **2014**, *5*, 3197–3209.

Table S5. All proteins differently expressed beween high and low secretome group. The proteomic comparison of enriched primary human AML cells showing high and low constitutive release of extracellular mediators; a summary for all proteins showing differential expression when comparing the two groups. The results are presented as the protein and gene names together the median levels for primary AML cells showing low of high constitutive mediator release, the relative fold change (log_2 scale), and the two right columns show the z-test p-value and the Welch's t-test p-value. Proteins with high expression in cells showing low constitutive release are marked with blue and high expression in cells with high constitutive release is indicated by red color.

Protein name	Gene name	Median level Low release	Median level High release	Relative difference (fold change) High versus low release	Z-test *p*-value (Fold Change significance)	Welch's *t*-test *p*-value Low versus high constitutive release
Spectrin beta chain. erythrocytic	SPTB	24.45	18.88	−5.57	0	0.042684
Ankyrin-1	ANK1	26.04	20.62	−5.42	0	0.042031
Extended synaptotagmin-2	ESYT2	23.81	20.94	−2.86	1.22×10^{-13}	0.0053
Histone H1.0; Histone H1.0. N-terminally processed	H1F0	27.70	25.30	−2.41	3.46×10^{-10}	0.018516
Alpha-synuclein	SNCA	25.78	23.54	−2.24	4.49×10^{-9}	0.015366
Succinate-semialdehyde dehydrogenase. mitochondrial	ALDH5A1	27.18	25.16	−2.02	1×10^{-7}	0.008151
SURP and G-patch domain-containing protein 2	SUGP2	24.77	22.92	−1.85	9.61×10^{-7}	0.03471
RNA-binding protein Musashi homolog 2	MSI2	24.33	22.50	−1.83	1.2×10^{-6}	0.012968
B-cell lymphoma/leukemia 11A	BCL11A	24.39	22.67	−1.72	4.67×10^{-6}	0.021886
AT-rich interactive domain-containing protein 1B	ARID1B	26.11	24.51	−1.60	1.75×10^{-5}	0.008946
7-dehydrocholesterol reductase	DHCR7	22.86	21.30	−1.56	2.79×10^{-5}	0.031664
Histone H2A type 1-J;Histone H2A type 1-H;Histone	HIST1H2AJ;HIST1H2AH;H2AFJ; HIST1H2AG;HIST1H2AD;HI	22.57	21.20	−1.37	0.000188	0.038121

H2A.J;Histone H2A type 1;Histone H2A type 1-D;Histone H2A type 1-A	ST1H2AA					
Metastasis-associated protein MTA1	MTA1	25.60	24.35	−1.25	0.000561	0.046264
Kinetochore-associated protein DSN1 homolog	DSN1	23.94	22.72	−1.22	0.000754	0.00714
Methyl-CpG-binding domain protein 3	MBD3	24.83	23.67	−1.16	0.001206	0.039855
Synaptotagmin-like protein 1	SYTL1	24.39	23.24	−1.15	0.001349	0.000491
ATP-dependent Clp protease ATP-binding subunit clpX-like. mitochondrial	CLPX	24.88	23.75	−1.13	0.001592	0.010674
Coiled-coil domain-containing protein 97	CCDC97	23.83	22.71	−1.12	0.001687	0.00485
Pogo transposable element with ZNF domain	POGZ	25.14	24.03	−1.10	0.001956	0.02357
DNA-directed RNA polymerase I subunit RPA1	POLR1A	25.49	24.42	−1.07	0.002519	0.010079
High mobility group protein 20A	HMG20A	24.77	23.70	−1.07	0.002578	0.017426
Rab-like protein 3	RABL3	23.51	22.44	−1.07	0.002628	0.002405
Core-binding factor subunit beta	CBFB	26.11	25.08	−1.04	0.003325	0.046175
DNA-directed RNA polymerase I subunit RPA2	POLR1B	24.10	23.09	−1.02	0.003823	0.020031
E3 ubiquitin-protein ligase RING2	RNF2	25.25	24.26	−1.00	0.00443	0.036123
55 kDa erythrocyte membrane protein	MPP1	25.50	24.52	−0.98	0.005	0.020847
Methylmalonyl-CoA mutase. mitochondrial	MUT	24.86	23.90	−0.97	0.005474	0.024224
Junctional adhesion molecule A	F11R	25.25	24.30	−0.95	0.006166	0.023361

Ashwin	C2orf49	23.50	22.55	−0.95	0.006402	0.001909
Cleavage and polyadenylation specificity factor subunit 4	CPSF4	24.15	23.21	−0.94	0.006581	0.005381
Chromodomain-helicase-DNA-binding protein 3	CHD3	22.95	22.01	−0.93	0.007103	0.019018
Glucosamine 6-phosphate N-acetyltransferase	GNPNAT1	23.27	22.36	−0.91	0.008097	0.030966
DNA-directed RNA polymerase I subunit RPA49	POLR1E	23.29	22.39	−0.91	0.008485	0.049055
Carbonic anhydrase 2	CA2	27.33	26.43	−0.91	0.008523	0.039685
Long-chain fatty acid transport protein 4	SLC27A4	22.25	21.36	−0.90	0.00914	0.007984
E3 SUMO-protein ligase PIAS1	PIAS1	22.86	21.98	−0.88	0.009831	0.003863
Poly [ADP-ribose] polymerase 1	PARP1	31.20	30.34	−0.86	0.011193	0.02295
Transcription factor jun-D	JUND	23.03	22.17	−0.86	0.011365	0.007308
Uroporphyrinogen decarboxylase	UROD	27.04	26.18	−0.86	0.011484	0.012351
Spermatid perinuclear RNA-binding protein	STRBP	25.94	25.09	−0.85	0.012156	0.044064
Cold-inducible RNA-binding protein	CIRBP	27.25	26.41	−0.83	0.01365	0.041227
Activator of basal transcription 1	ABT1	23.02	22.21	−0.81	0.01572	0.019734
Isobutyryl-CoA dehydrogenase. mitochondrial	ACAD8	23.43	22.62	−0.81	0.015852	0.041066
Ribosomal RNA processing protein 1 homolog B	RRP1B	24.97	24.16	−0.81	0.016278	0.025905
AP-1 complex subunit sigma-1A	AP1S1	22.59	21.79	−0.80	0.01668	0.023091
Actin-like protein 6A	ACTL6A	27.85	27.06	−0.79	0.017829	0.023698
7SK snRNA	MEPCE	24.73	23.95	−0.78	0.018871	0.025567

methylphosphate capping enzyme						
2-oxoisovalerate dehydrogenase subunit beta. mitochondrial	BCKDHB	25.75	24.97	−0.78	0.019368	0.039685
Uncharacterized protein C7orf50	C7orf50	23.81	23.05	−0.76	0.02186	0.02963
DNA-directed RNA polymerase I subunit RPA43	TWISTNB	22.41	21.66	−0.75	0.023213	0.02204
Prenylcysteine oxidase 1	PCYOX1	26.04	25.29	−0.74	0.023911	0.024481
SWI/SNF-related matrix-associated actin-dependent regulator of chromatin subfamily E member 1	SMARCE1	27.37	26.63	−0.74	0.024052	0.032658
Zinc finger protein 428	ZNF428	22.55	21.81	−0.74	0.024212	0.044007
Exosome complex component CSL4	EXOSC1	23.63	22.89	−0.74	0.02513	0.006291
PHD finger protein 3	PHF3	23.65	22.93	−0.71	0.028302	0.048744
Putative Dol-P-Glc:Glc(2)Man(9)GlcNAc(2)-PP-Dol alpha-1.2-glucosyltransferase;Dol-P-Glc:Glc(2)Man(9)GlcNAc(2)-PP-Dol alpha-1.2-glucosyltransferase	ALG10B;ALG10	23.06	22.35	−0.71	0.028778	0.003021
Ubiquitin carboxyl-terminal hydrolase 48	USP48	25.37	24.68	−0.70	0.031603	0.022852
Lanosterol synthase	LSS	23.97	23.29	−0.69	0.033442	0.023491
DNA repair protein XRCC1	XRCC1	25.73	25.04	−0.68	0.034084	0.033236
Inner nuclear membrane protein Man1	LEMD3	24.44	23.76	−0.68	0.034641	0.028543
Cytochrome b5	CYB5A	24.04	23.37	−0.68	0.035378	0.0346
Multidrug resistance-	ABCC1	22.69	22.02	−0.67	0.035616	0.023795

associated protein 1						
Exosome complex component RRP46	EXOSC5	25.38	24.72	−0.66	0.03881	0.048372
Scaffold attachment factor B2	SAFB2	25.87	25.21	−0.66	0.038908	0.0158
Vacuolar protein sorting-associated protein 72 homolog	VPS72	22.83	22.18	−0.65	0.041135	0.041684
Suppressor of SWI4 1 homolog	PPAN	22.62	21.97	−0.64	0.041982	0.009934
Endoplasmic reticulum metallopeptidase 1	ERMP1	27.36	26.72	−0.64	0.042459	0.026239
Menin	MEN1	23.45	22.81	−0.64	0.043127	0.039881
Cob(I)yrinic acid a.c-diamide adenosyltransferase. mitochondrial	MMAB	22.12	21.48	−0.64	0.043785	0.010839
Cleavage stimulation factor subunit 2 tau variant	CSTF2T	26.19	25.56	−0.63	0.045029	0.045067
Cip1-interacting zinc finger protein	CIZ1	21.70	21.07	−0.63	0.045352	0.049804
Nuclear receptor subfamily 2 group C member 2	NR2C2	22.69	22.06	−0.62	0.046622	0.025552
Lamin-B2	LMNB2	29.51	28.88	−0.62	0.04667	0.045373
Nucleoporin NUP53	NUP35	24.18	23.57	−0.61	0.049877	0.006672
Phosphatidylinositol 3.4.5-trisphosphate 5-phosphatase 1	INPP5D	27.20	27.97	0.76	0.047215	0.044399
RUN and FYVE domain-containing protein 1	RUFY1	26.03	26.79	0.77	0.047148	0.007889
Actin-related protein 3	ACTR3	30.03	30.79	0.77	0.046977	0.029739
Mitochondrial Rho GTPase 1	RHOT1	24.17	24.93	0.77	0.046607	0.02986
CD97 antigen;CD97 antigen subunit alpha;CD97 antigen subunit beta	CD97	26.57	27.34	0.77	0.046517	0.010544

Vesicle-associated membrane protein 3	VAMP3	25.18	25.95	0.77	0.046301	0.003763
Xaa-Pro dipeptidase	PEPD	25.74	26.51	0.77	0.046016	0.040364
NF-kappa-B essential modulator	IKBKG	23.53	24.31	0.77	0.045093	0.031763
Proteolipid protein 2	PLP2	24.56	25.34	0.79	0.042541	0.042301
Calcium-binding mitochondrial carrier protein SCaMC-1	SLC25A24	27.26	28.05	0.79	0.042528	0.017884
Dynamin-2	DNM2	21.34	22.13	0.79	0.041668	0.016485
Epsin-1	EPN1	22.96	23.76	0.79	0.040906	0.006474
Rho guanine nucleotide exchange factor 1	ARHGEF1	26.61	27.42	0.81	0.037731	0.024791
Vacuolar protein sorting-associated protein 13C	VPS13C	26.46	27.28	0.82	0.036679	0.016595
Rho GTPase-activating protein 27	ARHGAP27	22.36	23.18	0.82	0.035273	0.01106
Plexin-B2	PLXNB2	25.00	25.85	0.84	0.032126	0.004193
Integrin alpha-L	ITGAL	26.77	27.61	0.84	0.03177	0.011871
Serine/threonine-protein kinase TBK1	TBK1	22.65	23.50	0.85	0.031434	0.040984
Ras-related C3 botulinum toxin substrate 1;Ras-related C3 botulinum toxin substrate 3	RAC1;RAC3	25.46	26.32	0.86	0.029868	0.041908
Hematopoietic lineage cell-specific protein	HCLS1	28.22	29.08	0.87	0.028429	0.001312
Collagen type IV alpha-3-binding protein	COL4A3BP	23.42	24.29	0.87	0.027182	0.026718
Drebrin-like protein	DBNL	26.89	27.76	0.87	0.027079	0.021184
Coronin-7	CORO7	27.09	27.97	0.87	0.027074	0.027031
Nuclear factor NF-kappa-B p105 subunit;Nuclear factor NF-kappa-B p50 subunit	NFKB1	25.67	26.56	0.89	0.025365	0.006843
Alpha-1.3-mannosyl-	MGAT1	22.64	23.53	0.89	0.024897	0.032622

glycoprotein 2-beta-N-acetylglucosaminyltransferase						
SRSF protein kinase 2;SRSF protein kinase 2 N-terminal;SRSF protein kinase 2 C-terminal	SRPK2	21.52	22.42	0.90	0.023523	0.008372
Prosaposin;Saposin-A;Saposin-B-Val;Saposin-B;Saposin-C;Saposin-D	PSAP	28.04	28.94	0.90	0.02298	0.038796
Deoxyribose-phosphate aldolase	DERA	24.59	25.50	0.91	0.022803	0.018412
MOB kinase activator 3A	MOB3A	23.96	24.86	0.91	0.022685	0.045519
Rho GTPase-activating protein 9	ARHGAP9	24.58	25.49	0.91	0.022128	0.027527
Pro-interleukin-16;Interleukin-16	IL16	26.21	27.12	0.91	0.021885	0.040415
NADH dehydrogenase [ubiquinone] 1 alpha subcomplex assembly factor 4	NDUFAF4	22.90	23.82	0.92	0.021398	0.04093
Formin-like protein 1	FMNL1	27.29	28.22	0.93	0.020268	0.012477
Toll-interacting protein	TOLLIP	24.21	25.13	0.93	0.02023	0.019808
Arfaptin-1	ARFIP1	22.75	23.68	0.93	0.019985	0.008823
Ribosomal protein S6 kinase alpha-3	RPS6KA3	26.33	27.27	0.94	0.018806	0.041111
Alpha-N-acetylgalactosaminidase	NAGA	24.29	25.24	0.95	0.018001	0.016504
NADPH--cytochrome P450 reductase	POR	25.38	26.33	0.95	0.017609	0.013909
V-type proton ATPase subunit C 1	ATP6V1C1	24.60	25.56	0.96	0.016699	0.043686
Stromal membrane-associated protein 2	SMAP2	24.70	25.67	0.97	0.016133	0.041711
Cytosolic non-specific	CNDP2	27.71	28.69	0.98	0.015037	0.019533

dipeptidase						
SH3 domain-containing kinase-binding protein 1	SH3KBP1	25.69	26.67	0.98	0.015021	0.002592
DnaJ homolog subfamily C member 13	DNAJC13	26.57	27.56	0.98	0.01467	0.038713
Tyrosine-protein kinase SYK	SYK	27.18	28.17	0.99	0.014238	0.028254
Protein S100-A4	S100A4	29.66	30.65	0.99	0.013936	0.021936
Major vault protein	MVP	27.56	28.55	1.00	0.013507	0.02029
Phosphatidylinositol-binding clathrin assembly protein	PICALM	25.79	26.80	1.02	0.011976	0.00578
Serine/threonine-protein kinase N1	PKN1	24.62	25.64	1.02	0.011951	0.045995
Arf-GAP domain and FG repeat-containing protein 1	AGFG1	23.56	24.58	1.02	0.011885	0.009616
Ubiquitin carboxyl-terminal hydrolase 3	USP3	22.13	23.16	1.03	0.011455	0.009169
Neuroblast differentiation-associated protein AHNAK	AHNAK	31.88	32.93	1.05	0.009867	0.011693
Allograft inflammatory factor 1	AIF1	25.93	26.99	1.05	0.009683	0.02728
Copine-8	CPNE8	21.15	22.22	1.07	0.008848	0.044482
Ribonuclease inhibitor	RNH1	29.17	30.24	1.07	0.008834	0.029158
Tryptophan--tRNA ligase. cytoplasmic;T1-TrpRS;T2-TrpRS	WARS	27.38	28.46	1.08	0.0082	0.007696
Target of Myb protein 1	TOM1	25.53	26.62	1.09	0.007641	0.021214
Rho-related GTP-binding protein RhoG	RHOG	27.40	28.49	1.09	0.007631	0.023656
Protein-tyrosine kinase 2-beta	PTK2B	24.62	25.72	1.10	0.007407	0.008023
Leupaxin	LPXN	24.69	25.79	1.10	0.007032	0.025493
Calpastatin	CAST	26.18	27.29	1.10	0.00703	0.025903
Arf-GAP with Rho-	ARAP1	25.03	26.15	1.13	0.006072	0.01367

GAP domain. ANK repeat and PH domain-containing protein 1						
Early endosome antigen 1	EEA1	24.54	25.66	1.13	0.006029	0.027903
V-type proton ATPase subunit d 1	ATP6V0D1	25.30	26.43	1.13	0.005817	0.013603
Neutral cholesterol ester hydrolase 1	NCEH1	23.43	24.57	1.13	0.005743	0.024341
Ig gamma-1 chain C region	IGHG1	26.10	27.24	1.14	0.005561	0.036807
Phosphoenolpyruvate carboxykinase [GTP]. mitochondrial	PCK2	25.61	26.76	1.14	0.005388	0.011307
Serine beta-lactamase-like protein LACTB. mitochondrial	LACTB	24.28	25.43	1.15	0.005138	0.019455
Ubiquitin-like-conjugating enzyme ATG3	ATG3	24.73	25.90	1.16	0.0047	0.009424
Opioid growth factor receptor	OGFR	24.17	25.34	1.17	0.004655	0.004815
Alpha-1-antichymotrypsin;Alpha-1-antichymotrypsin His-Pro-less	SERPINA3	26.64	27.81	1.17	0.004543	0.004647
G protein-coupled receptor kinase 6	GRK6	22.74	23.92	1.18	0.004294	0.024724
E3 ubiquitin-protein ligase CBL	CBL	24.55	25.73	1.18	0.004201	0.023884
Protein S100-A6	S100A6	26.90	28.09	1.19	0.003855	0.029083
Protein FAM49B	FAM49B	26.95	28.17	1.22	0.003202	0.027396
N-acetyl-D-glucosamine kinase	NAGK	26.90	28.13	1.23	0.002925	0.016932
Ras-related protein Rab-27A	RAB27A	26.08	27.38	1.31	0.001729	0.033639
Cathepsin Z	CTSZ	26.66	27.97	1.31	0.001692	0.001369
Phostensin	PPP1R18	24.54	25.85	1.32	0.001568	0.010525
AP-1 complex subunit gamma-like 2	AP1G2	23.24	24.57	1.33	0.0014	0.010375

Integrin alpha-L	ITGAL	20.76	22.11	1.34	0.001299	0.016526
Pleckstrin homology domain-containing family O member 2	PLEKHO2	24.35	25.71	1.35	0.001235	0.002878
G-protein-signaling modulator 3	GPSM3	23.05	24.40	1.35	0.001209	0.007015
Protein unc-93 homolog B1	UNC93B1	22.97	24.33	1.36	0.001191	0.005074
Protein S100-A10	S100A10	26.24	27.61	1.37	0.001101	0.016931
Beta-arrestin-2	ARRB2	25.42	26.79	1.37	0.001072	0.01648
Procollagen galactosyltransferase 1	COLGALT1	26.12	27.48	1.37	0.001069	0.03412
Sorting nexin-1	SNX1	24.59	25.96	1.37	0.001055	0.006134
CapZ-interacting protein	RCSD1	25.83	27.22	1.39	0.000926	0.029507
IgG receptor FcRn large subunit p51	FCGRT	21.91	23.31	1.40	0.000867	0.017551
Osteoclast-stimulating factor 1	OSTF1	26.27	27.71	1.44	0.000628	0.02374
Serpin B8	SERPINB8	25.30	26.75	1.45	0.000554	0.010419
Sorting nexin-27	SNX27	24.06	25.53	1.46	0.000509	0.003824
Rho GTPase-activating protein 30	ARHGAP30	23.15	24.61	1.47	0.000493	0.006914
Rab11 family-interacting protein 1	RAB11FIP1	23.84	25.33	1.49	0.000412	0.01493
Annexin A2;Putative annexin A2-like protein	ANXA2;ANXA2P2	31.17	32.66	1.49	0.000409	0.002328
MAP kinase-activated protein kinase 2	MAPKAPK2	21.82	23.31	1.49	0.000395	0.046492
FK506-binding protein 15	FKBP15	24.88	26.39	1.51	0.000354	0.010975
Phosphatidylinositol 3.4.5-trisphosphate-dependent Rac exchanger 1 protein	PREX1	24.06	25.58	1.52	0.000316	0.041008
Integrin beta-2	ITGB2	28.57	30.09	1.53	0.000299	0.017857
P2X purinoceptor 4	P2RX4	22.31	23.84	1.53	0.000295	0.042154
Dynein light chain Tctex-type 1	DYNLT1	23.03	24.57	1.54	0.000274	0.041506
Alpha-1-	SERPINA1	27.54	29.08	1.54	0.000257	0.037304

antitrypsin;Short peptide from AAT						
HLA class II histocompatibility antigen. DM beta chain	HLA-DMB	23.00	24.57	1.56	0.000213	0.021238
Pyridoxal kinase	PDXK	24.94	26.53	1.59	0.000175	0.042089
Hexokinase-2	HK2	23.44	25.05	1.61	0.000145	0.019488
DNA helicase MCM9	MCM9	22.31	23.95	1.64	0.000111	0.03988
PML-RARA-regulated adapter molecule 1	PRAM1	25.79	27.42	1.64	0.00011	0.035983
Absent in melanoma 1 protein	AIM1	22.50	24.14	1.64	0.000107	0.026961
Calpain-2 catalytic subunit	CAPN2	27.19	28.84	1.65	0.0001	0.005489
EF-hand domain-containing protein D2	EFHD2	25.98	27.66	1.67	7.95×10^{-5}	0.006606
Protein S100-A11;Protein S100-A11. N-terminally processed	S100A11	28.99	30.66	1.67	7.91×10^{-5}	0.009925
Inosine-5-monophosphate dehydrogenase 1	IMPDH1	24.04	25.74	1.71	5.78×10^{-5}	0.000292
GEM-interacting protein	GMIP	24.14	25.85	1.71	5.67×10^{-5}	0.022388
Sorting nexin-18	SNX18	21.86	23.57	1.71	5.58×10^{-5}	0.010733
Protein Niban	FAM129A	24.87	26.60	1.72	4.97×10^{-5}	0.048942
Ras GTPase-activating-like protein IQGAP1	IQGAP1	29.34	31.07	1.73	4.72×10^{-5}	0.003266
Placenta-specific gene 8 protein	PLAC8	23.02	24.76	1.74	4.37×10^{-5}	0.045865
GTPase IMAP family member 4	GIMAP4	24.59	26.34	1.75	3.83×10^{-5}	0.032074
GTPase IMAP family member 7	GIMAP7	22.46	24.21	1.76	3.63×10^{-5}	0.028552
Arachidonate 5-lipoxygenase-activating protein	ALOX5AP	24.82	26.62	1.80	2.37×10^{-5}	0.037157
Cathepsin S	CTSS	26.11	27.93	1.82	1.94×10^{-5}	0.008482

Unconventional myosin-If	MYO1F	27.16	28.99	1.83	1.84×10^{-5}	0.015164
Syntenin-1	SDCBP	23.04	24.87	1.83	1.76×10^{-5}	0.01435
Golgi-associated plant pathogenesis-related protein 1	GLIPR2	25.03	26.86	1.84	1.64×10^{-5}	0.007614
Coactosin-like protein	COTL1	27.65	29.49	1.84	1.52×10^{-5}	0.016551
Galectin-3	LGALS3	24.48	26.33	1.85	1.43×10^{-5}	0.008959
Retinoid-inducible serine carboxypeptidase	SCPEP1	24.25	26.12	1.87	1.16×10^{-5}	0.039998
Caspase-1;Caspase-1 subunit p20;Caspase-1 subunit p10	CASP1	25.81	27.68	1.87	1.12×10^{-5}	0.013103
High affinity immunoglobulin epsilon receptor subunit gamma	FCER1G	23.52	25.40	1.88	1.03×10^{-5}	0.014456
Adenosine deaminase CECR1	CECR1	25.75	27.64	1.89	1×10^{-5}	0.013525
Receptor expression-enhancing protein 5	REEP5	23.41	25.35	1.94	5.69×10^{-6}	0.003174
Ferritin light chain	FTL	27.49	29.46	1.97	4.07×10^{-6}	0.025378
Docking protein 2	DOK2	23.41	25.42	2.01	2.57×10^{-6}	0.026624
Apolipoprotein B receptor	APOBR	26.21	28.23	2.01	2.51×10^{-6}	0.008843
Protein FAM65B	FAM65B	21.64	23.66	2.02	2.3×10^{-6}	0.003139
Long-chain fatty acid transport protein 3	SLC27A3	23.81	25.85	2.04	1.99×10^{-6}	0.00393
TYRO protein tyrosine kinase-binding protein	TYROBP	23.34	25.38	2.05	1.77×10^{-6}	0.005545
Cytochrome b-245 light chain	CYBA	24.70	26.76	2.05	1.61×10^{-6}	0.006509
Leukocyte immunoglobulin-like receptor subfamily B member 2	LILRB2	22.15	24.24	2.09	1.1×10^{-6}	0.005469
Receptor-type tyrosine-protein phosphatase epsilon	PTPRE	22.68	24.78	2.10	9.55×10^{-7}	0.013468
Mini-chromosome	MCMBP	23.22	25.35	2.13	6.62×10^{-7}	0.036588

maintenance complex-binding protein						
Neutrophil cytosol factor 4	NCF4	24.47	26.67	2.20	3.03×10^{-7}	0.032204
Biliverdin reductase A	BLVRA	24.91	27.11	2.20	2.93×10^{-7}	0.01654
Phospholipase D3	PLD3	23.51	25.75	2.24	1.82×10^{-7}	0.000942
Cytoskeleton-associated protein 4	CKAP4	24.79	27.05	2.26	1.41×10^{-7}	0.027987
Protein FAM107B	FAM107B	22.08	24.37	2.29	9.92×10^{-8}	0.016325
Deoxynucleoside triphosphate triphosphohydrolase SAMHD1	SAMHD1	28.90	31.24	2.33	5.72×10^{-8}	0.006459
Voltage-gated hydrogen channel 1	HVCN1	23.95	26.29	2.34	5.31×10^{-8}	0.001412
Engulfment and cell motility protein 2	ELMO2	22.43	24.79	2.36	4.04×10^{-8}	9.89E-05
Type-1 angiotensin II receptor-associated protein	AGTRAP	23.57	25.93	2.36	3.95×10^{-8}	0.001303
Neutrophil cytosol factor 2	NCF2	25.45	27.82	2.37	3.46×10^{-8}	0.033195
Tyrosine-protein kinase Fgr	FGR	23.77	26.18	2.41	2.21×10^{-8}	0.030902
Annexin A5	ANXA5	28.25	30.66	2.41	2.1×10^{-8}	0.020175
A-kinase anchor protein 13	AKAP13	22.96	25.42	2.46	1.11×10^{-8}	0.001665
Protein EVI2B	EVI2B	23.71	26.23	2.53	4.59×10^{-9}	0.000574
Tyrosine-protein phosphatase non-receptor type substrate 1	SIRPA	22.71	25.25	2.54	3.96×10^{-9}	0.029358
Arachidonate 5-lipoxygenase	ALOX5	23.20	25.84	2.64	8.81×10^{-10}	0.032953
Tumor necrosis factor alpha-induced protein 2	TNFAIP2	21.94	24.65	2.72	3.05×10^{-10}	0.025666
Brain acid soluble protein 1	BASP1	23.84	26.63	2.79	1.04×10^{-10}	0.011457
Tyrosine-protein kinase HCK	HCK	23.48	26.34	2.86	3.64×10^{-11}	0.037511

V-type proton ATPase 116 kDa subunit a isoform 3	TCIRG1	23.91	26.80	2.89	2.29×10^{-11}	0.024482
BTB/POZ domain-containing protein KCTD12	KCTD12	25.21	28.13	2.92	1.42×10^{-11}	0.012589
Toll-like receptor 2	TLR2	23.12	26.12	3.00	3.6×10^{-12}	0.007197
Dihydropyrimidine dehydrogenase [NADP(+)]	DPYD	24.24	27.36	3.12	5.03×10^{-13}	0.030795
Integrin alpha-M	ITGAM	25.54	28.76	3.22	9.7×10^{-14}	0.026935
Sulfotransferase 1A4;Sulfotransferase 1A3	SULT1A4;SULT1A3	22.76	26.02	3.26	5.02×10^{-14}	0.036611
Ribonuclease K6	RNASE6	21.72	25.02	3.30	2.33×10^{-14}	4.49×10^{-5}
Pro-cathepsin H;Cathepsin H mini chain;Cathepsin H;Cathepsin H heavy chain;Cathepsin H light chain	CTSH	22.34	25.65	3.31	1.93×10^{-14}	0.035829
Thymidine phosphorylase	TYMP	25.03	28.45	3.42	2.66×10^{-15}	0.005569
Myeloid cell nuclear differentiation antigen	MNDA	27.17	30.74	3.57	0	0.013294
Phospholipase B-like 1;Phospholipase B-like 1 chain A;Phospholipase B-like 1 chain B;Phospholipase B-like 1 chain C	PLBD1	21.70	25.50	3.80	0	0.000662
Cytochrome b-245 heavy chain	CYBB	25.40	29.22	3.82	0	0.017764
Heme oxygenase 1	HMOX1	20.53	24.62	4.09	0	0.002634
Lysozyme C	LYZ	29.17	33.40	4.22	0	0.015745
Neutrophil cytosol factor 1;Putative neutrophil cytosol factor 1B;Putative neutrophil cytosol	NCF1;NCF1B;NCF1C	23.81	28.24	4.43	0	0.008745

factor 1C						
Protein THEMIS2	THEMIS2	22.31	26.75	4.43	0	0.001118
Gamma-interferon-inducible lysosomal thiol reductase	IFI30	24.39	28.82	4.43	0	0.004753
Galectin-10	CLC	22.09	26.54	4.45	0	0.020032
Protein S100-A9	S100A9	27.69	32.69	5.00	0	0.004781
Protein S100-A8;Protein S100-A8. N-terminally processed	S100A8	27.52	32.58	5.05	0	0.005576
Bone marrow proteoglycan; Eosinophil granule major basic protein	PRG2	20.91	26.10	5.19	0	0.035599
Prolow-density lipoprotein receptor-related protein 1;Low-density lipoprotein receptor-related protein 1 85 kDa subunit;Low-density lipoprotein receptor-related protein 1 515 kDa subunit;Low-density lipoprotein receptor-related protein 1 intracellular domain	LRP1	20.48	26.53	6.06	0	4.36×10^{-5}
